# Sex Diversity in Heart Failure Clinical Trials: Actions Speak Louder Than Words∗

**DOI:** 10.1016/j.jacadv.2023.100786

**Published:** 2023-12-06

**Authors:** Vanessa Blumer, Roberta C. Bogaev, Mona Fiuzat

**Affiliations:** a Inova Schar Heart and Vascular, Falls Church, Virginia, USA; b Abiomed, J&J MedTech, Danvers, Massachusetts, USA; c Division of Cardiology, Duke University Medical Center, Durham, North Carolina, USA

**Keywords:** clinical trials, diversity, sex

**Figure:**
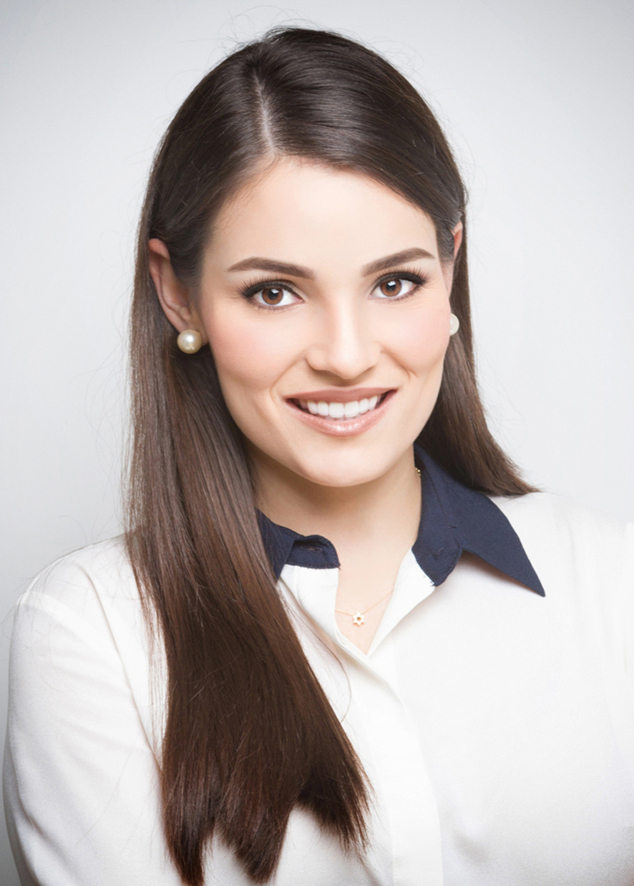

Despite substantial advancements in guideline-based medical therapies, heart failure (HF) persists as a major global health challenge, impacting millions worldwide including a high prevalence of women.^1^ Despite women being affected by HF more commonly than men across certain age groups and growing knowledge suggesting marked sex differences in presentation, clinical trajectory, response to therapy and outcomes, women patients remain underrepresented in clinical trials, and sex-specific data are lacking.^2^^,^^3^ Of note, throughout this editorial, we use the term sex to refer to the biological variable that is genetically determined (female/male), as opposed to gender, which is a multidimensional social construct (women/men). Since the vast magnitude of clinical studies rely on self-identified gender, we opted to use the terms women/men as a proxy for biological sex, while acknowledging that this has complex limitations.

In this issue of *JACC: Advances*, Ekpo et al^4^ present a thought-provoking study that adds to the growing body of evidence indicating that women are underrepresented in HF clinical trials. In their analysis, they specifically included HF clinical trials that observed mortality or hospitalization for HF, and they mirrored the timeline used by the 2022 American Heart Association/American College of Cardiology/Heart Failure Society of America Guidelines for the Management of Heart Failure,^5^ which included studies that were published through September 2021. Their final analysis comprised 33 randomized controlled trials with 104,972 trial participants; among these, only 23.2% were women (n = 24,366), with an overall participation prevalence ratio of 0.58 indicating significant underrepresentation. There was no change in representation over time, and underrepresentation was observed across all geographic regions of enrollment and trial funding type, though government-funded trials were significantly less representative than industry-funded trials.

The authors should be commended for their study, which once again highlights the well-documented lack of enrollment of women across HF clinical trials. The present study reiterates this disparity but, more importantly, should serve as a catalyst for change. Diversity is known to be a driver of innovation and excellence in medical research. However, the field of cardiology has yet to fully harness this potential, primarily due to the persistent underrepresentation of women.^6^ This gap not only hinders the development of more inclusive and effective HF care but also represents a missed opportunity for leveraging diverse perspectives that can lead to groundbreaking advancements in the field. Including women in HF trials is vital for several reasons. Diverse participant pools lead to more comprehensive data, ensuring that findings are applicable to a broader section of the population. This inclusivity also aids in identifying unique drug responses and potential side effects across different groups, thereby enhancing the safety and efficacy of medical therapies. Furthermore, the inclusion of women in HF trials can reveal valuable insights into sex-specific disease mechanisms and progression, potentially unveiling novel therapeutic targets. Ultimately, embracing sex diversity in HF research not only enhances the scientific rigor and relevance of the studies but also leads to more equitable health care outcomes, ensuring that both men and women benefit equally from the advancements in HF treatment and management.

The underrepresentation of women in HF clinical trials is a known and persistent problem.^2^^,^^6^ At this point, this matter demands more than acknowledgment; moving beyond mere words, it's time for immediate, deliberate, and action-oriented solutions. Effectively addressing these disparities requires a multifaceted approach, including the commitment from researchers, regulators, and industry to ensuring that women are adequately represented in all aspects of clinical research, from trial design to execution and analysis (Figure 1). Among possible solutions, it has been shown that enrollment of women in clinical trials is more likely to occur when female investigators are part of the trial leadership; as such, increasing the representation of female researchers is critical.^2^^,^^6^ With this in mind, providing targeted training and mentorship programs is important to help cultivate a diverse pool of skilled researchers that can subsequently lead to intentional recruitment of women in trials. The PLATINUM Diversity Trial serves as a paradigmatic example of effective strategies led by diverse researchers to overcome enrollment biases, demonstrating that through careful site selection and dedicated efforts, it is indeed feasible to enroll women and minorities in large-scale, national, multicenter outcome studies at rates comparable to those of traditional studies. This study successfully achieved a participant composition of approximately 50% women and significantly increased the representation of minority groups, doubling the typical proportions seen in similar studies.^7^ Importantly, the Food and Drug Administration had a recent policy change that underscores this commitment to diversity, whereby it requires sponsors to submit comprehensive plans for enrolling diverse patient groups, providing clear targets and strategies for achieving these goals.^8^ Similarly, industry sponsors should commit to setting enrollment targets based on the prevalence of disease in women, developing diversity action plans for each clinical trial in accordance with guidance from the Food and Drug Administration, tracking enrollment and retention of women throughout the study as well as regular reviews of a robust screen failure log to identify barriers for women to enroll and remain in clinical trials.

**Figure 1:**
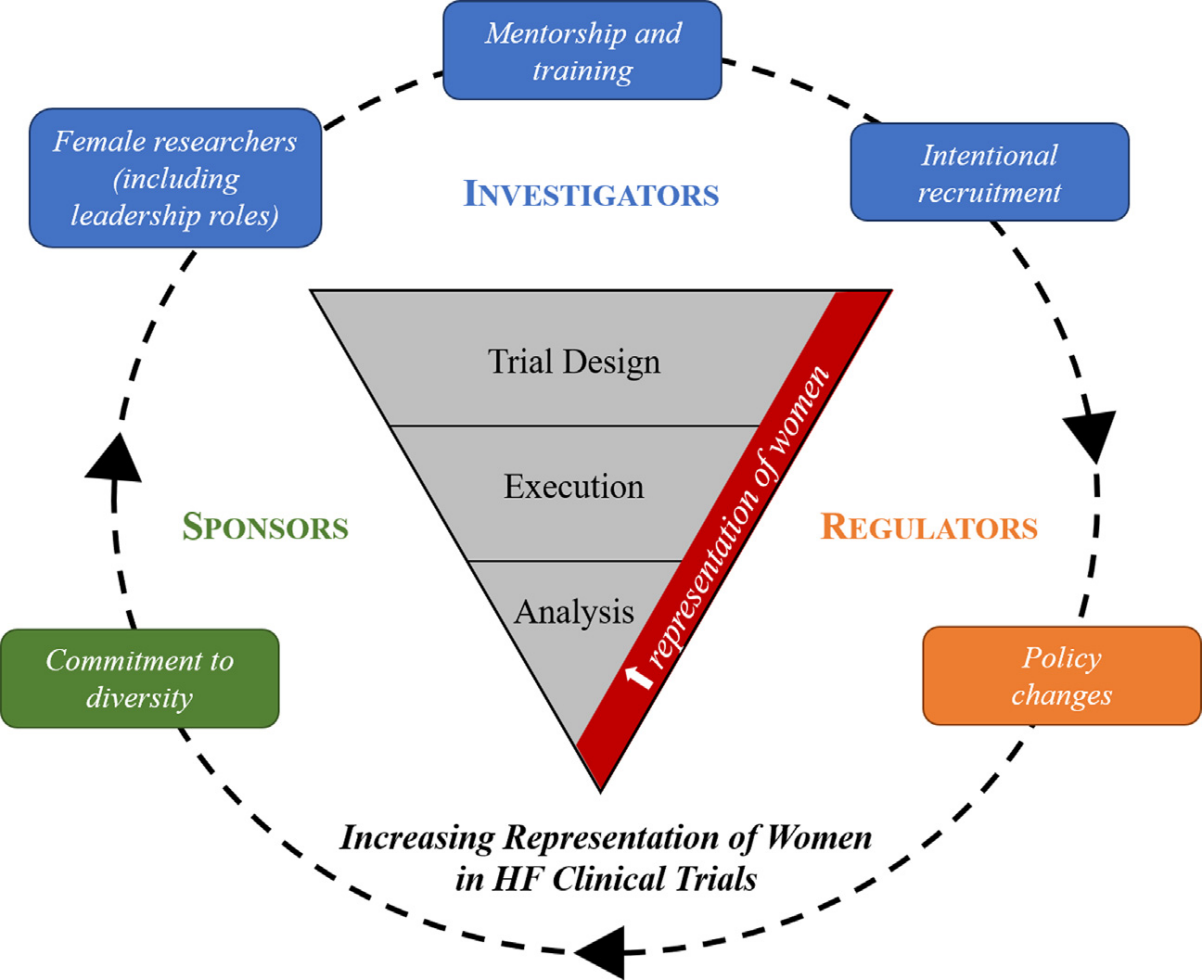
**Increasing Representation of Women in HF Clinical Trials** Effectively addressing disparities that lead to underrepresentation of women in HF clinical trials requires multifaceted interventions involving investigators, sponsors, and regulators. An action-based approach should include: increasing the pool and offering more opportunities to female researchers; providing targeted training and mentorship programs to cultivate a diverse pool of skilled female researchers; intentional recruitment of women participants; policy changes; and an overall commitment to diversity, ensuring that women are adequately represented in all aspects of clinical research, from trial design to execution and analysis. HF = heart failure.

The Heart Failure Collaboratory (HFC), a consortium dedicated to enhancing clinical trial efficiency and conduct, serves as a prime example of implementing effective, action-based solutions for promoting diversity in medical research.^9^ Acknowledging the critical role of diverse perspectives in scientific innovation, the HFC has proactively initiated strategies to cultivate a varied pool of investigators, including acknowledgment of diverse trial enrollment and diverse research teams through the Site-Based Research Awards. HFC’s initiatives are not limited to recruitment; they also encompass comprehensive training and mentorship programs aimed at nurturing a new generation of diverse clinical trialists. In a strategic move to influence the broader research landscape, the HFC has also collaborated with editors of leading HF journals, advocating for diversity in trial leadership and authorship. This engagement highlights the HFC's commitment to infusing diversity at all levels of clinical research. One of the HFC’s notable initiatives includes the establishment of a Data Safety Monitoring Board workshop, specifically designed to broaden the scope of participation among young and diverse professionals. This workshop focuses on equipping the next generation with the necessary skills and knowledge to assume roles in trial leadership, including steering committees and clinical events/adjudication committees. These multifaceted efforts by the HFC are a testament to the power of targeted actions in fostering diversity. By creating more inclusive platforms for research and leadership, the HFC is not only contributing to the immediate goal of diversified HF research but is also paving the way for a more inclusive future in medical science.

In conclusion, the study by Ekpo et al^4^ serves as a wake-up call to the medical research community about the ongoing sex disparity in HF clinical trials. While the problem is clear, the solutions require concerted efforts from all stakeholders, including researchers, industry sponsors, and regulatory bodies. As we move forward, it is crucial to translate our understanding of this issue into action, ensuring that clinical research in HF and beyond truly reflects and serves the diverse population it aims to heal. The future of equitable and effective medical therapy depends on our ability to embrace and implement these changes.

## Funding support and author disclosures

Dr Bogaev serves as vice president for heart failure at Abiomed, Inc. All other authors have reported that they have no relationships relevant to the contents of this paper to disclose.
